# Minimally Invasive Surgery in Pediatric Surgical Oncology

**DOI:** 10.3390/children5120158

**Published:** 2018-11-26

**Authors:** Hannah M. Phelps, Harold N. Lovvorn

**Affiliations:** 1 School of Medicine, Vanderbilt University, Nashville, TN 37232, USA; 2 Department of Pediatric Surgery, Vanderbilt University Medical Center, Nashville, TN 37232, USA; harold.lovvorn@vanderbilt.edu

**Keywords:** minimally invasive surgery, pediatric cancer, neuroblastoma, Wilms tumor, rhabdomyosarcoma

## Abstract

The application of minimally invasive surgery (MIS) to resect pediatric solid tumors offers the potential for reduced postoperative morbidity with smaller wounds, less pain, fewer surgical site infections, decreased blood loss, shorter hospital stays, and less disruption to treatment regimens. However, significant controversy surrounds the question of whether a high-fidelity oncologic resection of childhood cancers can be achieved through MIS. This review outlines the diverse applications of MIS to treat pediatric malignancies, up to and including definitive resection. This work further summarizes the current evidence supporting the efficacy of MIS to accomplish a definitive, oncologic resection as well as appropriate patient selection criteria for the minimally invasive approach.

## 1. Introduction

Adult colon cancer represents the first application of minimally invasive surgery (MIS) for definitive resection of malignant disease. The use of MIS for definitive resection of adult cancers has expanded rapidly over the past 15 years, and increasing types and numbers of intrabdominal malignancies are being treated with MIS, including the complexities of pancreatic adenocarcinoma [1,2]. For any operative approach, an oncologic resection that includes complete removal of tumor with negative margins, adequate lymphadenectomy, and preservation of adjacent organs when possible is the primary goal. Among appropriately selected adult patients, the minimally invasive approach has been shown to be oncologically equivalent to open resection for a variety of malignancies, including gastric cancer, colon cancer, and liver cancers [3,4,5]. With growing application of MIS for cancer operations in adults, the technique has emerged as a treatment option for pediatric cancer as well. To date, however, the efficacy of and appropriate patient selection for MIS remain less well defined in pediatric surgical oncology than in adults.

### Fundamental Questions Surrounding MIS Resection of Pediatric Cancer

1. How does the minimally invasive approach affect the ability to achieve a complete or gross total resection, negative margin status, and adequate lymph node sampling for a given tumor type?
2. How does MIS impact relapse-free and overall survival?
3. What patient and tumor characteristics should be considered during patient selection?
4. What are the technical considerations?

## 2. History of MIS for Treating Pediatric Malignancy

**Figure 1:**
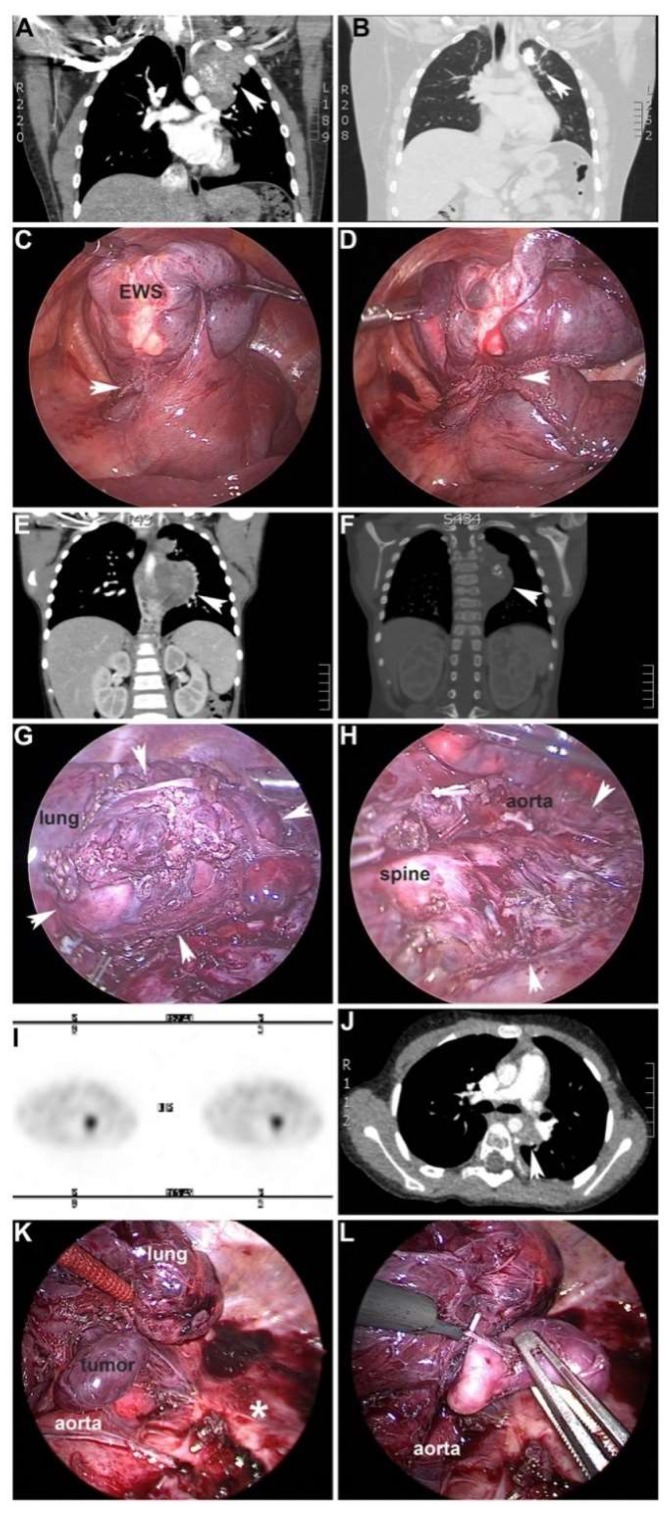
Minimally invasive surgery (MIS) resection of thoracic pediatric embryonal tumors. (**A**–**C**) Adolescent male who was diagnosed with a primary Ewing sarcoma (EWS) of the left upper pulmonary lobe (**A**; arrowhead). Marked regression of primary lesion was observed after neoadjuvant therapy (**B**; calcified nodule and arrowhead). (**C**) Thoracoscopic view of superior segment of left upper lobe and mass (EWS). Arrowhead denotes staple line after dividing segmental artery to mass. (**D**) Completing the segmentectomy with a linear stapler. Arrowhead denotes resection staple line. (**E**–**H**) Four-year-old girl who presented with a large left-sided thoracic neuroblastoma (NBL). (**E**,**F**) Mass before and after neoadjuvant therapy (arrowhead). (**G**,**H**) Thoracoscopic resection of large thoracic NBL. (**G**) Borders of the mass after mobilization are depicted with arrowheads. Collapsed lung is labeled. Note the profound angiogenic nature of NBL and desmoplastic response after neoadjuvant therapy. (**H**) Tumor bed after complete resection. Arrowheads depict cephalad and caudal borders of tumor bed. (**I**–**L**) Three months after completing therapy, Metaiodobenzylguanidine (MIBG)-avidity of a hilar lymph node persisted (**I**; dark spot). Arrowhead denotes mass on CT scan (**J**). (**K**,**L**) Repeat thoracoscopic approach to resect hilar “tumor” 12 months after initial operation. Lung and aorta are labeled. Asterisk denotes primary tumor bed free of local relapse (**K**). (**L**) Hook cautery dissection of dumbbell-shaped hilar mass in atraumatic grasper.

Early uses of MIS in treating pediatric cancers were limited to biopsy, staging, evaluation of resectability, and management of therapeutic complications, such as infection [6]. With time, MIS has been utilized for definitive resection of pediatric malignancies. An analysis of five years of experience at the St. Jude Children’s Research Hospital applying MIS in the comprehensive care of pediatric cancer patients revealed that a total of 64 laparoscopic and 49 thoracoscopic procedures were performed on 101 patients during the study period (1995–2000). Of the 99 successful MIS procedures, 53 diagnosed or evaluated disease, 32 managed complications of therapy, 7 removed metastatic deposits, and 7 resected primary tumors (2 splenectomies, 2 oophorectomies, 2 adrenalectomies, and 1 partial hepatectomy) [7]. Over the past 15 years, MIS has increasingly been applied for definitive resection of solid tumors in children, particularly for neuroblastoma (Figure 1) [8,9,10,11,12,13,14]. Recent reports have also described the utility of MIS to resect Wilms tumor either through a nephron-sparing approach or complete nephroureterectomy [8,15,16]. Table 1 outlines some of the larger studies to report on the use of MIS in the treatment of pediatric cancers.

## 3. Efficacy of MIS to Treat Pediatric Malignancy

With the growing interest in using MIS to resect pediatric solid tumors, the question emerged whether a high-fidelity oncologic resection could be achieved in children. In other words, how does the minimally invasive approach affect the ability to achieve a complete or gross total resection, negative microscopic margins, and adequate lymph node sampling? And what is its impact on perioperative outcomes, relapse-free and overall survival? To date, no randomized controlled clinical trials have been conducted to compare MIS to open surgery for resecting pediatric solid tumors. Such studies are likely not feasible given the often massive tumors presenting in small children and disease heterogeneity [18]. After an attempted multi-institutional, prospective, randomized controlled trial was terminated early due to lack of patient accrual, investigators created a survey to assess contributing factors. Interestingly, 40% of responding surgeons reported discomfort with the technical aspects of a minimally invasive oncologic resection. Notably, family preference did not appear to be a major limitation, as parents of all eligible children who were approached agreed to participate. The investigators concluded that the study failed for a variety of reasons, including delay in distribution of protocols to investigators, failure to submit protocols for Institutional Review Board (IRB) approval, surgeon discomfort with MIS, and surgeon bias toward either a minimally invasive or open approach [19]. Several cohort studies have described institutional experiences with MIS, but few have commented on the oncologic integrity attainable with this approach (Table 2) [10,12]. Even fewer have commented on appropriate patient selection for a MIS. Due to the lack of clear evidence regarding patient selection, potential benefits, and the oncologic integrity of a minimally invasive resection, we recently conducted an in-depth analysis of our institutional experience using MIS to resect embryonal tumors [14]. We hypothesized that, among appropriately selected patients, MIS can maintain oncologic integrity while minimizing interruptions to therapy.

### 3.1. Oncologic Integrity

Our recent analysis is the first report that provides an in-depth critique of MIS to achieve gross total resection (GTR, >98% resection of a neuroblastic lesion assessed by postoperative imaging), negative microscopic margins, and adequate lymph node sampling as well as its impact on relapse-free (RFS) and overall survival (OS) [14]. The largest tumor resected with MIS in this cohort measured 100 mL. Thus, to control for tumor size, we compared only outcomes for tumors measuring less than 100 mL at the time of open or MIS resection. Most importantly, we found that RFS and OS were not compromised with the minimally invasive approach. Specifically, for tumor volumes <100 mL, the five-year RFS was 0.90 (95% CI, 0.66–0.97) after MIS and 0.77 (95% CI, 0.64–0.86) after open resection (*p* = 0.249). Five-year OS was 1.00 (95% CI, 1.00–1.00) after MIS and 0.80 (95% CI, 0.67–0.89) after open resection (*p* = 0.124). Furthermore, high rates of GTR were achieved with MIS (94%, *n* = 16). Also, no significant difference in margin status was observed between tumors resected open or with MIS (*p* = 0.333). Finally, the median number of lymph nodes sampled with MIS was 1 (interquartile range (IQR) 0, 3) compared to 2 (IQR 1, 5.5) lymph nodes harvested with an open approach (*p* = 0.070) [14]. Though not statistically significant, this potential difference indicates that care must be taken to ensure adequate lymph node sampling with a minimally invasive approach. An analysis of the National Cancer Database provides a less granular but larger-scale analysis that supports these findings [20]. No significant difference was observed in surgical margin status or one-year and three-year survival when comparing MIS (*n* = 1330) to open resection (*n* = 141) of neuroblastoma and Wilms tumors. In that report, however, a higher rate of lymph node evaluation and a greater number of total lymph nodes sampled were documented when using an open approach [21]. Importantly, oncologic goals differ for neuroblastoma, Wilms tumor, and other embryonal tumors. For example, GTR is a lower priority for neuroblastoma as the data indicate that as little as 50% resection is adequate for low-risk tumors. While lymph node harvest is required for staging of both neuroblastoma (International Neuroblastoma Staging System) and Wilms tumor, the lymph node status is considered more prognostically significant in Wilms tumor.

### 3.2. Patient Selection

**Figure 2:**
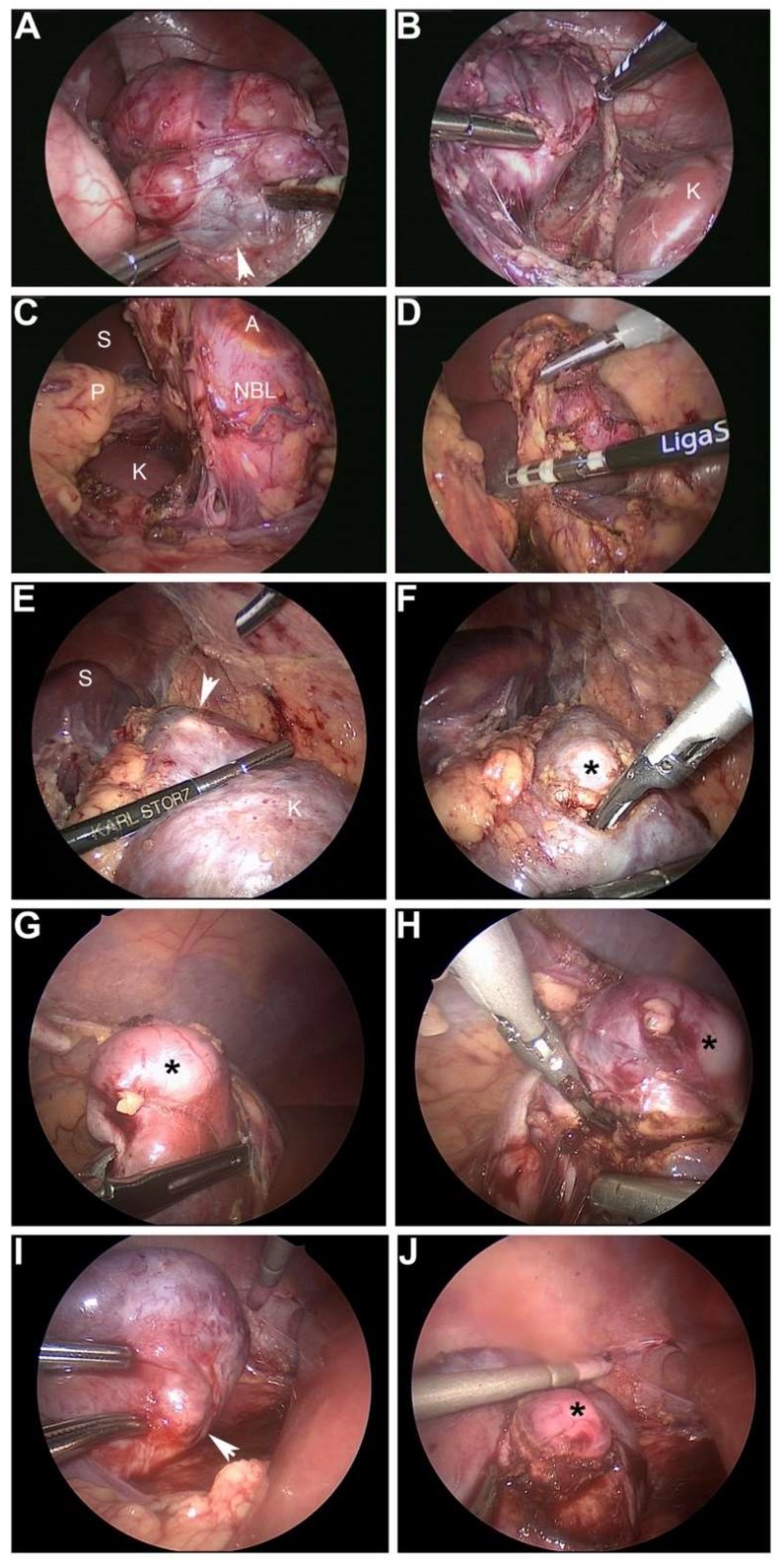
MIS resection of neuroblastoma and Wilms tumor. (**A**,**B**) 20-month-old male who was diagnosed with a left adrenal neuroblastoma secreting vasoactive intestinal polypeptide. Arrowhead shows confluence of adrenal vein (draped across mass) with left renal vein (**A**). (**B**) Dissection of NBL away from left kidney (labeled K). (**C**,**D**) Infant who presented with opsoclonus-myoclonus and was discovered to have a left adrenal NBL (labeled; A, adrenal; S, spleen; P, pancreas; K, kidney). Complete resection with negative margins was achieved in both cases using a bipolar energy vessel sealer. (**E**,**F**) 18-month-old male with Beckwith–Wiedemann syndrome who was discovered on routine cancer screening to have a left renal mass consistent with Wilms tumor. Images show resection with ultrasonic scalpel of residual mass in left upper pole after six weeks of neoadjuvant therapy. Arrowhead and asterisk denote 1 cm mass (S, spleen). (**G**–**J**) Four-year-old girl who had been treated in infancy for diffuse hyperplastic perilobar nephrogenic rests. On routine cancer screening, two right renal cortical masses consistent with Wilms tumor were discovered. (**G**,**H**) MIS resection of lower pole Wilms tumor (asterisk) with ultrasonic scalpel. (**I**,**J**) Resection of right upper pole Wilms tumor, also with ultrasonic scalpel (arrowhead and asterisk).

Patient selection is of utmost importance when considering MIS for tumor resection in children. However, few authors have detailed specific selection criteria of the ideal patient amenable to a minimally invasive approach. Often, the decision is based on surgeon comfort with the approach to a given case rather than strict or objective criteria. One report suggested that neuroblastic tumors lacking image-defined risk factors (IDRF; e.g., vascular encasement, intraspinal tumor extension, infiltration into adjacent organs) were feasible to a minimally invasive approach [13]. One other institution defined similar criteria for laparoscopic resection of neuroblastoma as diameter <5 cm in the largest dimension and absence of vascular encasement [12]. Our recent analysis also identified tumor size (as estimated through radiographic measurements) and IDRF as two key factors in patient selection. Tumors that were selected for MIS all had tumor volumes less than 100 mL. Though embryonal tumors are characteristically quite large at presentation, we noted specific circumstances in which volumes less than 100 mL were encountered. Specifically, tumors that were remarkably responsive to neoadjuvant chemotherapy, presented early in the context of paraneoplastic symptoms (including opsoclonus-myoclonus ataxia and vasoactive intestinal polypeptide-secreting tumors), or were detected in children under active surveillance for a cancer-predisposing syndrome, such as hemihypertrophy, Beckwith–Wiedemann, or WT1 mutations, often had volumes less than 100 mL (Figure 2). All but one of the neuroblastic tumors resected minimally invasively had zero IDRF at time of resection. Finally, MIS resections were more commonly performed for neuroblastic tumors compared with other types of embryonal tumors [14].

### 3.3. Benefits of MIS

The same benefits of MIS that apply when treating benign diseases theoretically can be realized in the malignant context as well, including decreased postoperative pain, lower incidence of postoperative intestinal ileus, reduced hospital stays, and earlier return to activity. Indeed, studies have shown that MIS for pediatric cancer is associated with decreased length of hospital stay, decreased blood loss, and decreased time to initiation of chemotherapy after laparoscopic biopsy [10,12,22]. Our findings also indicated that MIS resection is associated with decreased blood loss, shorter hospital stay, and decreased operating time. Furthermore, our results showed that the median time after resection to the next chemotherapy was 19.5 days (IQR 14, 26) in children undergoing open surgery with tumor volume less than 100 and 12.5 days (IQR 7.5, 19.5) in children undergoing MIS (*p* < 0.051) [14]. Though not statistically significant, a trend toward sooner initiation of adjuvant chemotherapy after a minimally invasive resection was found. The question of whether MIS offers quicker return to strict chemotherapy timelines merits further investigation with a larger sample size.

## 4. Additional Applications of MIS

Though this review focuses on the utility of MIS for definitive resection of pediatric *embryonal* tumors, other applications of MIS in treating pediatric cancers should also be noted. For example, video-assisted thoracoscopic surgery (VATS) for pulmonary metastasectomy in the setting of osteosarcoma has been recommended based on the finding that patients presenting with single pulmonary lesions on CT did not have additional nodules detected at the time of thoracotomy [23]. Thus, it is currently considered safe and potentially beneficial to resect thoracoscopically a *single* pulmonary metastatic deposit detected on high-resolution CT in those patients who may require multiple thoracic operations. More recently, uniportal VATS has been employed for excisional biopsy of peripheral lung nodules in pediatric cancer patients [24]. A variety of image-guided techniques have been proposed for localization, including intrathoracosopic ultrasound and CT-guided needle localization with methylene blue staining [23]. A prospective clinical trial to assess VATS versus thoracotomy for resection of osteosarcoma pulmonary metastases is currently under consideration by the Children’s Oncology Group. However, for diffuse, bilateral metastatic deposits, palpation via thoracotomy provides the optimal approach to identify and resect all calcified nodules that are concerning for disease.

When lymph node dissection is indicated, a laparoscopic approach may be suitable in the appropriately selected patient. For example, laparoscopic retroperitoneal lymph node dissection has been described for high-risk (age greater than 10 years) pediatric patients with paratesticular rhabdomyosarcoma. Based on a small retrospective case series, the authors concluded that the approach is a safe diagnostic and therapeutic procedure [25].

Finally, while ovarian masses are commonly resected minimally invasively, an open approach is preferred for ovarian malignancy to ensure proper Children’s Oncology Group (COG) staging of germ cell tumors, which involves visual inspection and physical palpation of ovaries, omentum, and peritoneum. Differentiating benign and malignant disease preoperatively is complex and lacks definitive criteria. Tumor characteristics favoring malignancy include size ≥8 cm, presence of thick septations >2–3 mm and nonhyperechoic solid nodular or papillary components, and tumor markers (LDH, AFP, β-HCG, and CA-125). The absence of tumor markers does not exclude malignancy, and the presence of tumor makers, particularly LDH and AFP, does not mandate malignancy. Thus, tumor markers must be assessed within the context of other clinical characteristics [26,27].

## 5. Limitations of MIS to Treat Pediatric Malignancy

With increasing application of MIS in the malignant context, its technical and oncologic limitations should also be considered carefully. From a technical standpoint, often large tumors must be mobilized within small spaces, and tactile constraints and reduced visibility may limit the efficacy of a minimally invasive approach. Individual surgeon experience with MIS also contributes to the ability to effectively mobilize tumors with vascular encasement and other IDRF without compromising nearby structures or causing tumor spillage. Challenges unique to MIS include difficulty with vascular control when dissecting tumors away from large vessels and maintaining adequate pneumoperitoneum to visualize tissue planes when aspirating angiogenic bleeding.

Specific concerns about how iatrogenic pneumoperitoneum or pneumothorax might affect tumor spread and additional concerns about the potential for port site recurrence have contributed to the controversy surrounding the use of MIS when treating solid malignancies in children. In one murine model of neuroblastoma, mice undergoing carbon dioxide pneumoperitoneum demonstrated a higher rate of hepatic metastases but not local peritoneal spread compared to mice undergoing laparotomy [28]. Some evidence suggests that carbon dioxide incubation promotes increased protein expression of certain proto-oncogenes, including C-MYC and its target, HGMB-1 [29]. Such adverse effects of carbon dioxide pneumoperitoneum have not been documented in humans.

Port-site recurrence has been documented in MIS resection of adult cancers and represents another manner in which a less invasive approach may uniquely affect disease outcomes [30]. Such occurrences have not been observed in pediatric patients undergoing MIS for a cancer operation [31]. Several of the reports included in this review comment specifically on the absence of port-site recurrence within their respective cohorts [14,17].

## 6. Technical Considerations: Pearls and Pitfalls of the MIS Approach

### 6.1. Technical Pearls

1. Identify appropriate patient and cancer type.
2. Leverage neoadjuvant chemotherapy to shrink tumors to facilitate resection when appropriate (e.g., no vasvular encasement and manageable tumor volume).
3. Position patient for success.
4. Commit to the challenge of completing procedure with MIS.
5. Memorize preoperative imaging and location of vascular and other vital structures (and display images during procedure for frequent reference).
6. Must achieve the appropriate oncologic principles for the specific tumor type.
  - Complete or gross total (>98%) resection of neuroblastoma. *
  - Complete resection of Wilms tumor without spill and adequate lymph node sampling.
7. Have laparoscopic suction in field for short bursts to aspirate angiogenic bleeding, especially if resecting a tumor after neoadjuvant therapy.
8. Recommend ultrasonic scalpel when excising mass from kidney or liver to preserve margin analysis given less thermal spread. Bipolar vessel sealers are adequate as well if greater distance from specimen is permissible and when dividing larger vessels.
9. Surgical clips or vascular staplers are good for dividing larger vessels.
10. Monitor progress regarding time, blood loss, and oncologic integrity. Be prepared to open if absolutely necessary.
11. Use a specimen bag to remove the tumor.
12. Identify a port location having good cosmesis to deliver specimen in a bag.
13. Lengthen this ideal port to the narrowest diameter of tumor and deliver in that dimension.

* Note: The value of radical surgery for neuroblastoma is controversial. As little as 50% resection is sufficient in patients with low-risk disease. For intermediate-risk disease, the goal is to achieve the most complete resection possible with minimal morbidity. The COG high-risk protocol currently recommends gross total resection with removal of locoregional disease, despite conflicting evidence on the role of surgery in high-risk neuroblastoma [32]. Regardless, resection extent should not be sacrificed in favor of a minimally invasive approach.

### 6.2. Technical Pitfalls

1. Inappropriate patient selection:
  - Excessively large tumors.
  - Vascular encasement.
  - Limited tactile feedback, or haptics, with MIS instruments to appreciate large vessels, so must have good visualization of critical structures.
2. Not mentally committing to MIS approach. These procedures are challenging.
3. Not positioning patient appropriately.
4. Tissue planes are difficult after neoadjuvant therapy (desmoplasia is challenging to dissect) and if angiogenic bleeding is excessive.
5. Not recognizing feeding and draining vessels (i.e., difficult to control large anatomic vessels with MIS, so need to know location precisely).
6. Not sampling lymph nodes adequately.
7. Not completing the appropriate oncologic operation for a given tumor type.
8. Recommend using a specimen bag.
9. Do not morcellate tumor to deliver specimen—it must remain intact.

## 7. Conclusions

As MIS is increasingly applied to the resection of pediatric malignancies, the questions of appropriate patient selection and oncologic integrity with this approach remain controversial. Despite emerging evidence suggesting that MIS achieves oncologic equivalence to open resections in terms of RFS, OS, GTR, and margin status, no prospective randomized trial exists to confirm this notion because the need for careful patient selection precludes randomization. Currently, the characteristic tumor amenable to a minimally invasive resection appears most commonly to be a neuroblastic tumor without IDRF and with a tumor volume less than 100 mL. It is expected that the range of tumors considered to be amenable to MIS will broaden with increasing surgeon comfort using this approach, as is evident from the adult literature [1]. Continued improvement in neoadjuvant therapies and screening protocols of children genetically predisposed to develop an embryonal tumor may render more cases amenable to MIS resection. Therefore, understanding the impact of this approach on tumor biology and patient outcomes is critical. As always, maintaining oncologic principles remains the priority when considering any approach.

## Figures and Tables

**Table 1:** Reports on MIS for pediatric cancers.

Study	Total Procedures	Intent	Conversions	Complications
Spurbeck 2004 [7]	64 laparoscopy	27 diagnosis/evaluation 7 resection 1 Hodgkin’s disease 1 CML 1 ALL 1 large-cell lymphoma 1 ganglioneuroma 1 pheochromocytoma 1 mesothelioma 30 treatment of complication	4	2 liver hematoma 1 bowel injury
	49 thoracoscopy	7 evaluation 40 biopsy/resection of pulmonary lesion 2 treatment of complication	14	2 intraoperative desaturation 1 intraoperative bleeding
Metzelder 2007 [9]	65 laparoscopy	41 biopsy/staging 24 resection 6 NBL 1 lymphoma 3 ovarian cancer 4 suspicious liver lesions 2 suspicious kidney lesions 8 suspicious lesions, other	16	1 bowel injury 2 intraoperative bleeding
	25 thoracoscopy	14 biopsy/staging 11 resection 3 NBL 1 lymphoma 1 lung metastasis 6 unknown	5	1 intraoperative bleeding
Leclair 2008 [17]	45 laparoscopy	45 resection 45 NBL	4	1 bowel obstruction due to entrapment in trocar orifice 1 ischemia of kidney 1 wound abscess
Malek 2010 [10]	11 thoracoscopy	11 resection 11 NBL	0	2 Horner syndrome 1 severe atelectasis
Fraga 2012 [11]	17 thoracoscopy	17 resection 17 NBL	0	2 Horner syndrome
Kelleher 2013 [12]	18 laparoscopy	18 resection 18 NBL	2	None reported
Warmann 2014 [16]	24 laparoscopy	24 resection 24 WT	0	1 splenic injury
Irtan 2015 [13]	19 laparoscopy	19 resection 19 NBL	0	1 renal atrophy
	20 thoracoscopy	2 biopsy 2 NBL 18 resection 18 NBL	3	1 Horner syndrome 3 chylothorax
Phelps 2018 [14]	17 laparoscopy	17 resection 13 NBL 3 WT 1 RMS	0	No acute complications
	8 thoracoscopy	8 resection 8 NBL	0	No acute complications
	1 cystoscopy	1 resection 1 RMS	0	No acute complications

CML, chronic myelogenous leukemia; ALL, acute lymphoblastic leukemia; NBL, neuroblastic tumor; WT, Wilms tumor; RMS, rhabdomyosarcoma.

**Table 2:** Evaluation of MIS as a tool for oncologic resection.

Citation	MIS Resections	Conversions	GTR	Negative Margins	Lymph Nodes	Median Follow-Up	Relapse and Survival
Spurbeck 2004 [7]	7	0/7	NR	NR	NR	NR	NR
Metzelder 2007 [9]	35	14/35 (40%)	NR	NR	NR	39 mo	NR
Leclair 2008 [17]	45	4/45 (9%)	43/45 (96%)	37/45 (82%)	NR	28 mo	OS: 84% ± 8.1 EFS: 77% ± 9.1 1 local + metastatic relapse 1 local relapse 1 metastatic relapse 1 progressive disease
Malek 2010 [10]	11	0/11	NR	3/7 (43%)	NR	NR	EFS: 9.1% OS: 100% 1 local relapse
Fraga 2012 [11]	17	0/17	17/17 (100%)	17/17 (100%)	NR	16 mo	OS & EFS: 100%
Kelleher 2013 [12]	18	2/18 (11%)	NR	NR	NR	L/I risk: 42 mo H risk: 19 mo	L/I risk: 5-yr EFS and OS 100% H risk: numbers too small to calculate, 1 death
Warmann 2014 [16]	24	0/24	24/24 (100%)	21/24 (88%)	15/24 (63%) sampled	47 mo	EFS: 95.8% OS: 100%
Irtan 2015 [13]	37	3/37 (8%)	32/37 (86%)	NR	NR	25 mo	5-yr OS: 97.7% 5-yr EFS: 97.7% 1 metastatic relapse
Phelps 2018 [14]	26	0/26	17/18 (94%)	9/20 (45%)	6/26 (23%) sampled	58 mo	5-yr RFS: 0.90 (CI, 0.66–0.97) 5-yr OS: 1.00 (CI, 1.00–1.00)

NR, not reported; GTR, gross total resection (as defined by the author); EFS, event-free survival; OS, overall survival; L/I risk, low/intermediate risk; H risk, high risk.
